# Measurement of Transient Overvoltages by Capacitive Electric Field Sensors

**DOI:** 10.3390/s24051357

**Published:** 2024-02-20

**Authors:** Felipe L. Probst, Michael Beltle, Stefan Tenbohlen

**Affiliations:** Institute of Power Transmission and High Voltage Technology (IEH), University of Stuttgart, 70569 Stuttgart, Germany; michael.beltle@ieh.uni-stuttgart.de

**Keywords:** capacitive electric field sensor, capacitive divider, high-voltage measurement system, switching transients, transient overvoltages

## Abstract

The accurate measurement and the investigation of electromagnetic transients are becoming more important, especially with the increasing integration of renewable energy sources into the power grid. These sources introduce new transient phenomena due to the extensive use of power electronics. To achieve this, the measurement devices must have a broadband response capable of measuring fast transients. This paper presents a capacitive electric field sensor-based measurement system to measure transient overvoltages in high-voltage substations. The concept and design of the measurement system are first presented. Then, the design and concept are validated using tests performed in a high-voltage laboratory. Afterwards, two different calibration techniques are discussed: the simplified method (SM) and the coupling capacitance compensation (CCC) method. Finally, three recorded transients are evaluated using the calibration methods. The investigation revealed that the SM tends to overestimate the maximum overvoltage, highlighting the CCC method as a more suitable approach for calibrating transient overvoltage measurements. This measurement system has been validated using various measurements and can be an efficient and flexible solution for the long-term monitoring of transient overvoltages in high-voltage substations.

## 1. Introduction

Transient overvoltage events are one of the major causes of dielectric failures in substation equipment [1,2]. These events are caused by switching operations, lightning discharges, and short circuits, among others [3]. With the increasing integration of renewable energy sources into the electric power system, the system dynamics are changing, and new transient phenomena are occurring [4,5,6]. Investigating their effects on substation equipment and accurately measuring the transients becomes even more important.

The required frequency bandwidth of the measuring device depends on the transient being measured. In high-voltage substations, voltage measurement is often carried out using capacitive voltage transformers (CVT) or inductive voltage transformers (IVT). Although these devices provide accurate measurements at nominal frequency, their outputs deviate significantly at higher frequencies [7,8,9,10,11]. Consequently, this characteristic restricts their application for measuring fast transients.

To overcome the limitations of voltage transformers, specific techniques have been proposed in the literature [10,11,12,13,14,15,16]. Most of these methods focus on correcting the distorted output voltage. However, these approaches often require complex filtering techniques or the installation of additional devices. Another strategy to measure transients is to use voltage sensors connected to the measurement tap of transformer bushings [17,18,19,20,21]. While this technique has high accuracy and wide bandwidth, its use is limited to transformer bays. 

Using electric field sensors for voltage measurement is another solution that has been the subject of research and further improvement [22,23,24,25,26,27,28,29,30,31,32,33]. In [25,26,27,28], the authors propose voltage calculation through inversion or integration of the measured electric field. However, these methods require complex algorithms to solve matrix inverse operations or numerical integration of the spatial electric field. This results in low accuracy and long computation time [25,28]. In [29], the voltage is calculated from the rate of change in the electric displacement field, using a differential self-integration D-dot sensor. However, this sensor is a near-field device positioned on the ceramic casing of the conductor, exhibiting inflexibility and a potential galvanic risk. Another investigated technique involves voltage measurement based on a differentiating/integrating (D/I) concept [30,31,32], utilizing a capacitive pick-up electrode composed of a parallel plate capacitor with a grounded bottom plate. The primary drawback of this method is its low accuracy due to the neglect or inaccurate estimation of coupling between the outer phases. In [33], various alternative methods for measuring transients using unconventional transducers are discussed. One of these methods employs a coupling plane as an electric field sensor; however, the author provides only a brief overview of the technique. 

This paper presents a measurement system based on capacitive electric field sensors to measure transient overvoltages in a high-voltage substation. Two prior publications [34,35] have briefly outlined the system’s concept, major components, and its field application. In [34], the measurement system monitored switching transient overvoltages during the energization of a 420 kV transmission line. The primary aim was to investigate the failure of a surge arrester during a switching operation. In [35], the authors evaluated some measured transient overvoltages in the frequency domain. The analysis revealed that the transients contain significant high-frequency components (>>1 kHz), making traditional IVT or CVT unsuitable for accurately measuring them. 

This study extensively discusses the concept and design of the measurement system, introduces an improved calibration method, and provides a statistical analysis of signals measured during a one-year measurement campaign. Section 2 outlines the system’s concept and design. Section 3 reports the results of the validation performed in a high-voltage laboratory. Section 4 describes the aspects of the measurement system installation in a high-voltage substation and discusses two calibration methods. Section 5 presents the statistical analysis of all recorded transients and evaluates three of them using the different calibration methods. Finally, Section 6 presents the conclusions of the study.

## 2. Concept and Design of the Measurement System

**Figure 1:**
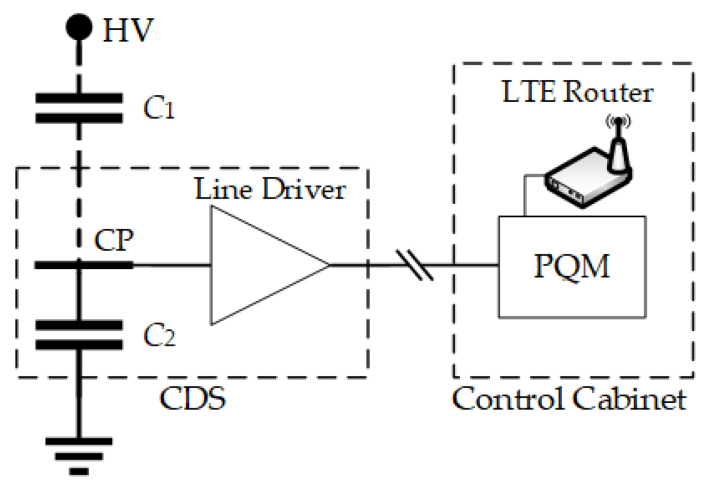
Schematic diagram of the transient overvoltage measurement system.

A measurement system based on capacitive electric field sensors has been developed to measure transient overvoltages in substations. Figure 1 shows its schematic diagram.

**Figure 2:**
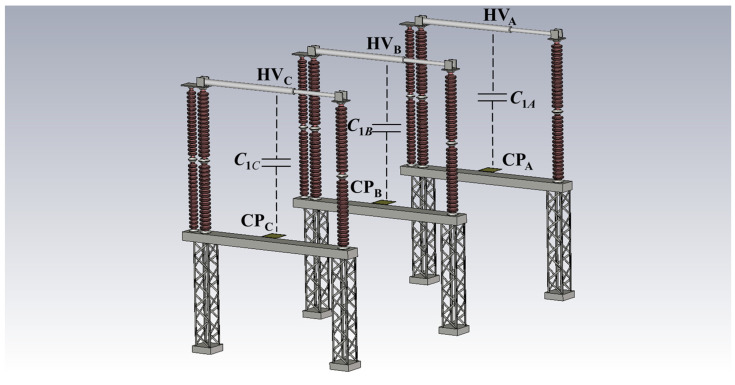
Three-dimensional model of the measurement setup for calculation of stray capacitance *C*_1_.

The capacitance *C*_1_ represents the stray capacitance between the coupling plane (CP) and the high-voltage connection (HV) if the measurement device is installed under a substation busbar or on the base of a disconnector (see Figure 2). The user-defined capacitance *C*_2_ is connected between CP and ground and is chosen based on the desired voltage ratio. There is also a stray capacitance between the CP and ground (around 30 pF), but it is much smaller than *C*_2_ and can be neglected. Therefore, considering both capacitances *C*_1_ and *C*_2_, the measurement system has a capacitive divider whose voltage ratio is given by:

(1) $$\frac{V_{in}}{V_{out}}=\frac{C_{1}+C_{2}}{C_{1}}$$

where *V_in_* is the voltage between HV and ground, and *V_out_* is the divider’s output voltage.

Equation (1) is used to calculate the expected voltage ratio, considering the maximum voltage expected in a transient overvoltage (*V_in-max_*) and the maximum output voltage suitable for the measurement system (*V_out-max_*). In this study, *V_in-max_* is considered as 1 MV and *V_out-max_* as 20 V. Thus, the theoretical voltage ratio is 50,000:1. To accurately determine this voltage ratio, the capacitances *C_1_* and *C_2_* must be known. However, *C*_1_ is a stray capacitance that changes according to the layout where the measurement system is installed. Therefore, this capacitance is estimated using the finite element method (FEM) in CST Studio Suite^®^ version 2021 [36]. The three-dimensional model of the setup is shown in Figure 2.

The three sensors are installed, one on each base of a three-phase disconnector. The electric field can be assumed to be quasi-static considering the steady-state voltage. As a result, it exhibits the characteristics of an electrostatic field [25]. Therefore, the stray capacitance *C*_1_ is calculated using the electrostatic solver in CST Studio.

The CST Studio model uses the technical drawing of the substation as the reference. The distance between phases is 4.50 m. The disconnector base is made of metal and has a height of 2.75 m. The moving contact of the disconnector is 3.75 m long and has a radius of 0.05 m. The insulators are modeled as ceramic porcelain with a relative permittivity ε = 6, a relative permeability μ = 1, a height of 3.35 m, and an outer radius of 0.13 m. The sensor’s CP is 0.10 m above the base of the disconnector. It has a length, width, and height of 0.40 m, 0.40 m, and 0.04 m, respectively. All the metal parts are modeled as perfect electric conductors (PEC), with the voltage of the moving contact set at 242.5 kV. The sensors and bases are grounded.

According to the electrostatic simulation, the calculated capacitances, *C*_1*B*_, and *C*_1*C*_ are 0.766 pF, 0.772 pF, and 0.776 pF, respectively. The difference between *C*_1*A*_ and *C*_1*C*_ arises from the inherent approximation within the FEM. To achieve a voltage ratio of 50,000:1, the capacitance *C*_2_ should be 38.6 nF, based on the average of the calculated *C*_1_ values. Additionally, there is a coupling between each sensor and the adjacent phases. This topic will be discussed in Section 4.

### 2.1. The Capacitive Divider Setup

**Figure 3:**
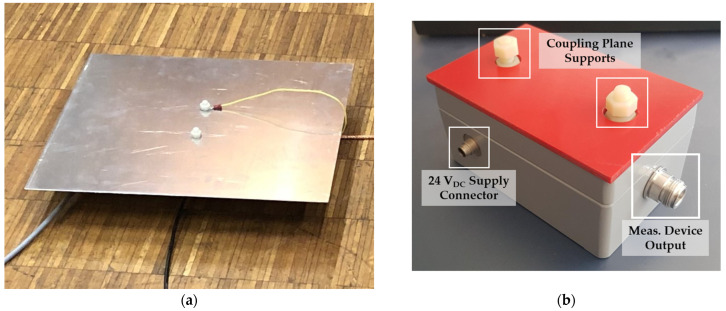
External components of the CDS. (**a**) Coupling plane; (**b**) metal housing with connections.

After estimating the capacitance *C*_1_ and defining the voltage divider requirements, the capacitive divider setup (CDS) is designed and assembled. The CDS comprises a CP and an electronic circuit mounted in an IP65 metal enclosure. The CP is an aluminum plate connected to the electronic circuit through an external terminal. Figure 3 shows the CP (a) and the metal housing (b) of the electronic circuit. The housing has four external connectors: one 4 mm connector for the CP, one N-connector for the measurement coaxial cable, one waterproof 2-pin connector for the DC source, and one ground connector.

Inside the metal housing, the CP connects to the capacitors constituting *C*_2_. Specifically, six 8.3 nF temperature-stable SMD capacitors are connected in parallel to achieve a capacitance of 49.8 nF. If the output of the voltage divider is directly connected to a measurement instrument using a long coaxial cable, the cable’s capacitance influences the voltage ratio of the measurement system. Consequently, a line driver is employed to decouple the capacitive divider from the coaxial measurement cable.

**Figure 4:**
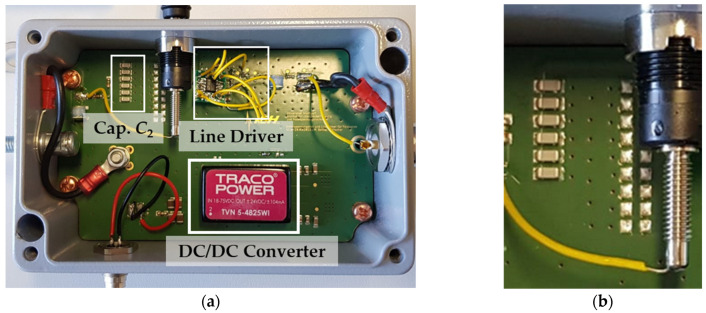
Electronic circuit of the CDS. (**a**) Top view; (**b**) parallel capacitors of *C*_2_.

The line driver uses a JFET input operational amplifier [37] with a slew rate of 60 V/µs, a bandwidth of 14 MHz, and a supply voltage of ±20 V. The circuit board also has additional internal buffered converters [38] to ensure voltage stability. A 15 m coaxial cable connects the line driver output to a power quality monitor. Figure 4 shows the electronic circuit of the CDS (a) and the six parallel capacitors of *C*_2_ (b).

### 2.2. The Power Quality Monitor

A power quality monitor [39] is installed in a control cabinet to read and record the measured transient signals. It measures high-frequency harmonics and transients at a sampling rate of 1 MS/s. It has eight channels with synchronous sampling: four channels with 4 mm connectors with an input impedance of 10 MΩ and an input range from −600 V to +600 V; and four channels with BNC connectors with an input impedance of 1 MΩ and an input range from −50 V to +50 V. The transients recorded by the power quality monitor PQM-800 are stored internally and can also be uploaded to a monitoring cloud. 

**Figure 5:**
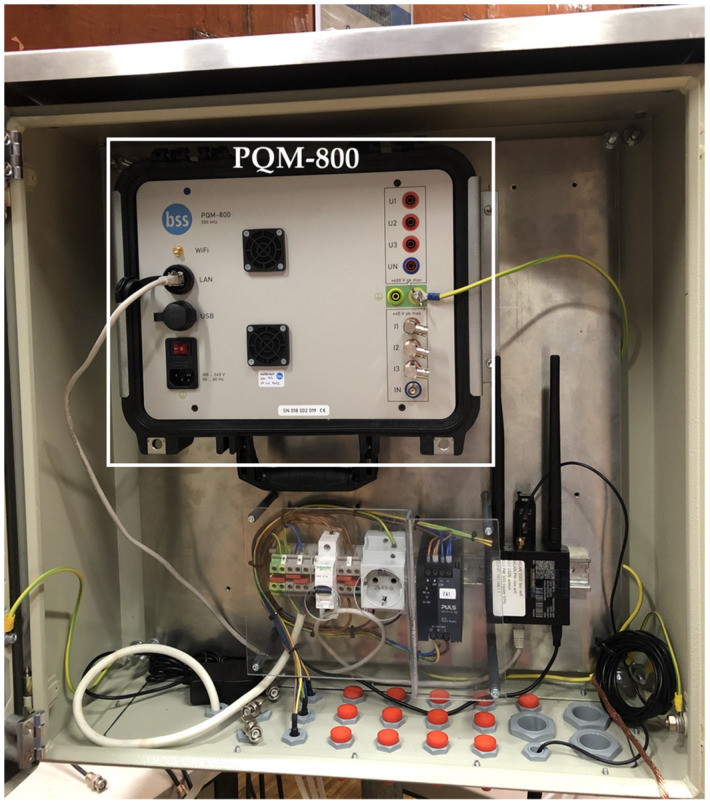
The control cabinet with the power quality monitor.

The control cabinet also includes an LTE router for sending the recorded transients to the monitoring cloud and a 24 V DC power supply for the CDS, as shown in Figure 5.

## 3. Tests in the High-Voltage Laboratory

Three CDSs are assembled for monitoring transient overvoltages in the substation. Prior to installation, the devices are tested in the high-voltage laboratory of the University of Stuttgart, where the following tests are conducted:

- Measurement of *C*_2_ capacitances;
- Accuracy assessment (measurement of divider ratio and stray capacitances *C*_1_);
- Lightning impulse test;
- Measurement of the CDS bandwidth.

The capacitances *C*_2_ of the CDSs, formed by temperature-stable multilayer ceramic capacitors (MLCC), are measured using a multimeter. The values obtained for the devices MD1, MD2, and MD3 are 49.94 nF, 50.76 nF, and 50.53 nF, respectively.

### 3.1. Accuracy Assessment Using AC Voltage

**Figure 6:**
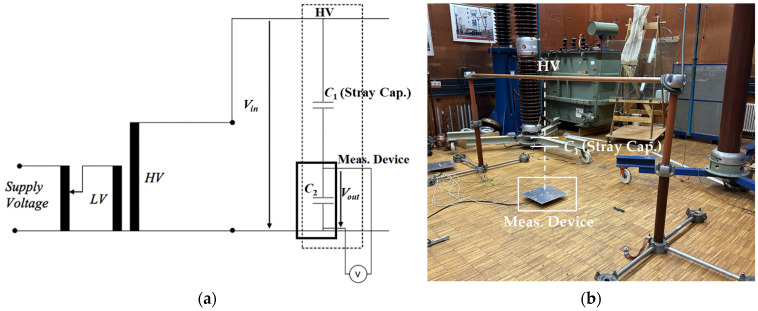
Measurement of the accuracy of the CDSs. (**a**) Schematic diagram; (**b**) test setup.

The accuracy of the three CDSs is assessed by measuring the voltage ratio and the stray capacitance *C*_1_ of the setup built in the high-voltage laboratory. Despite differences from the configuration in Figure 2, it is still possible to evaluate the linearity of the measurement system response since *C*_1_ is the only parameter that changes based on the layout. The measurements are performed individually with an input voltage range from 13 kV_rms_ to 76 kV_rms_. Figure 6 shows the schematic diagram (a) and the setup (b) for measuring the accuracy of the CDSs.

**Figure 7:**
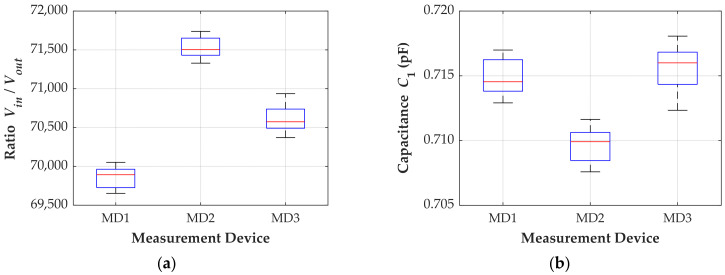
Measurement results. (**a**) Voltage ratio; (**b**) stray capacitance *C*_1_.

The input high voltage *V_in_* is measured using a standard capacitive divider with a ratio of 3060:1. Both signals, the output of the standard divider and the output of the CDSs (*V_out_*), are evaluated with a 70 MHz oscilloscope with a sampling rate of 1 GS/s. The stray capacitance *C*_1_ is then calculated from Equation (1) using the measured values of *C*_2_, *V_in_*, and *V_out_*. Figure 7 shows the measured values of voltage ratio (a) and stray capacitance *C*_1_ (b). Ten measurements are conducted for each device, with the input voltage ranging from 13 kV_rms_ to 76 kV_rms_.

The median voltage ratios for devices MD1, MD2, and MD3 are 69,892:1, 71,503:1, and 70,574:1, respectively. The average voltage ratios are 69,859:1, 71,535:1, and 70,610:1. Distinct voltage ratios are expected due to the dependence of the ratio on *C*_1_ and *C*_2_ and the different capacitances *C*_2_ among the CDSs.

The stray capacitance *C*_1_ should ideally have a unique value for a given configuration. However, variations are observed in the measurements of the three units, potentially attributed to sensor sensitivity or slight misalignment of the devices during measurements. Considering the average value of *C*_1_ for each unit, the maximum error observed is 0.29% for devices MD1 and MD2, and 0.46% for device MD3.

The accuracy of the measurement devices is assessed by analyzing the voltage ratio measured for each device. The ratio error is calculated as follows:

(2) $$\varepsilon =\frac{{(k}_{r}\;V_{out}-V_{in})\;}{V_{in}}\times \;100\%$$

where *k_r_* is the average of the measured voltage ratios for each device. The results are given in Table 1, which shows a linear behavior of the measurement devices concerning the applied voltage. The maximum error observed is 0.29% for devices MD1 and MD2, and 0.46% for device MD3, relative to their average voltage ratios.

### 3.2. Lightning Impulse Test

**Figure 8:**
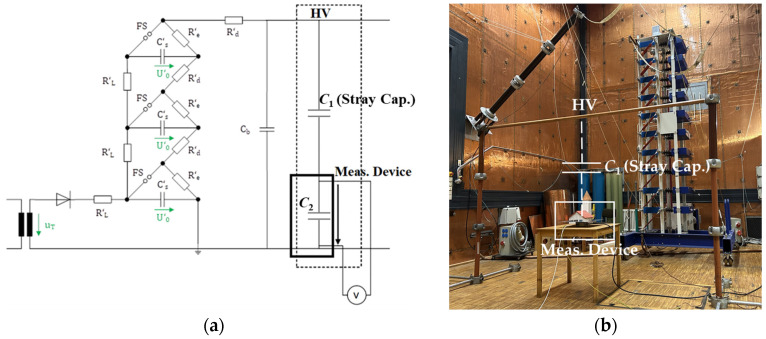
Lightning impulse test. (**a**) Schematic diagram; (**b**) test setup.

The lightning impulse test is the next evaluation performed in the high-voltage laboratory. The standard IEC 60060-1 [40] defines a lightning impulse as a double exponential waveform characterized by a front time *T*_1_ of 1.2 µs ±30% and a tail time *T*_2_ of 50 µs ±20%. The impulse generator can deliver switching and lightning impulse voltages up to 1 MV and a total energy of 30 kJ. Figure 8 illustrates the schematic diagram (a) and test setup (b).

**Figure 9:**
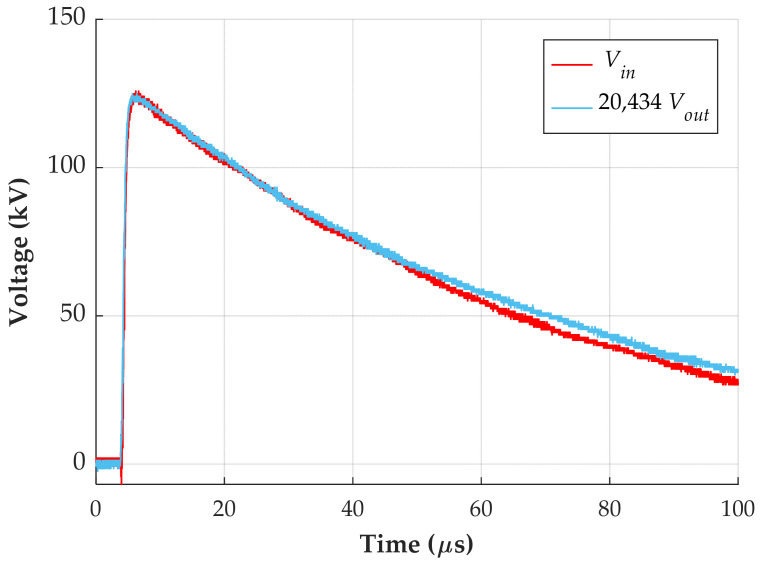
Lightning impulse response of device MD1.

The applied impulse voltage is measured using a standard capacitive divider. Its output and the output from device MD1 are evaluated using an oscilloscope with a bandwidth of 1 GHz and a sampling rate of 10 GS/s. Figure 9 shows the measurement of the lightning impulse, where the output voltage from unit MD1 is multiplied by the voltage ratio measured for this configuration, which is 20,434:1.

The applied lightning impulse has a front time of 1.22 µs and a tail time of 51.48 µs, whereas device MD1 exhibits a front time of 1.17 µs and a tail time of 54.35 µs. Both signals meet the criteria of IEC 60060-1 and demonstrate similarity. Therefore, the measurement device can accurately reproduce a lightning impulse signal.

### 3.3. Measurement of the CDS Bandwidth

**Figure 10:**
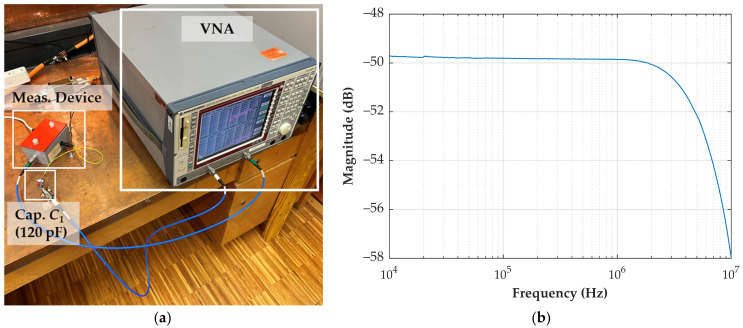
Bandwidth measurement of device MD1. (**a**) Test setup; (**b**) measurement result.

The measurement of the CDS bandwidth is performed using a 120 pF ceramic capacitor to simulate the stray capacitance *C*_1_. Although the typical stray capacitance is less than 1 pF, a higher value is chosen to improve the signal-to-noise ratio of the measurement. Using a Rohde & Schwarz ZVRE vector network analyzer (VNA) [41] with a frequency range from 9 kHz to 4 GHz, the measurement assessed the *V_out_*/*V_in_* ratio over a frequency range spanning from 10 kHz to 10 MHz. Figure 10 shows the test setup (a) and the obtained measurement results (b).

The frequency response measurement shows a constant ratio of −49.8 dB up to 1.2 MHz, with the 3 dB frequency at 5.6 MHz. It indicates that the measurement device has a broadband response and can measure fast transients. However, the power quality monitor limits the bandwidth of the measurement system to a frequency range of up to 500 kHz, which is still sufficient to measure fast transients.

## 4. Installation and Calibration

**Figure 11:**
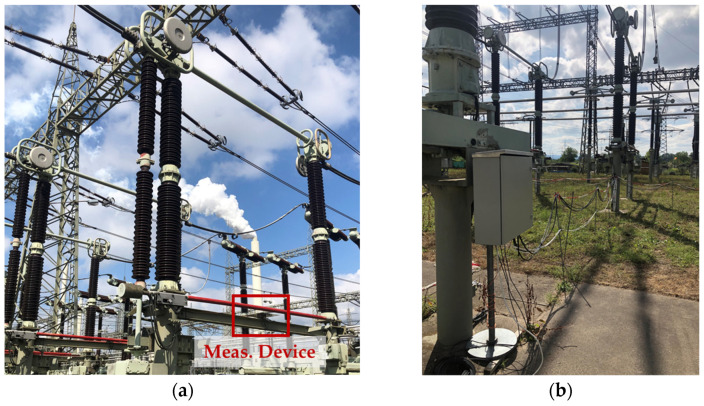
Measurement system installed in the substation. (**a**) CDS on the base of a disconnector; (**b**) Control cabinet on the concrete base of a circuit breaker.

The measurement system was installed in a high-voltage substation for a one-year measurement campaign. The CDSs were mounted on the bases of a three-phase disconnector in a transmission line bay. Devices MD1, MD2, and MD3 were installed in phases A, B, and C, respectively. The control cabinet was placed on the concrete base of a circuit breaker, approximately six meters from the devices. Figure 11 shows a CDS (a) and the control cabinet (b) installed in the substation.

**Figure 12:**
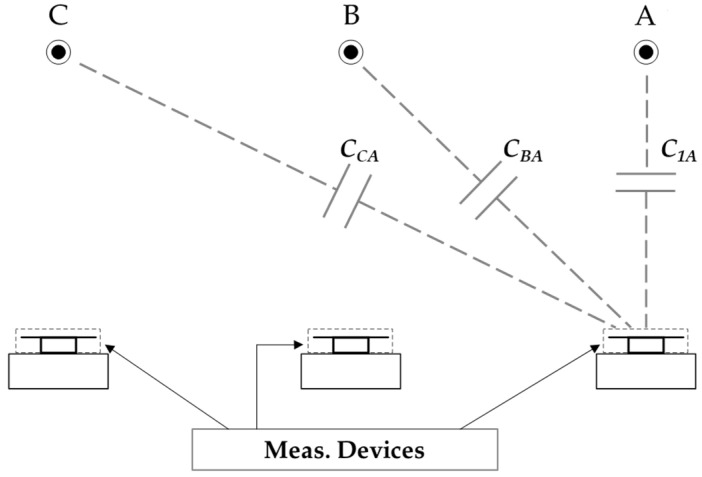
Coupling capacitances between a CDS and adjacent phases.

The value of the stray capacitance *C*_1_ depends on the distance and layout. Additionally, there is a coupling between the adjacent phases and the sensor, influencing its output voltage *V_out_*, as illustrated in Figure 12.

Figure 12 shows only the coupling capacitances between the CDS installed in phase A and the three high-voltage connections. The same happens with the CDSs installed in phases B and C. To mitigate cross-coupling effects in the measurement, it is necessary to calibrate the measurement system. Two calibration techniques are examined:

- Simplified method (SM);
- Compensation of coupling capacitances (CCC).

### 4.1. Calibration Using the Simplified Method

**Figure 13:**
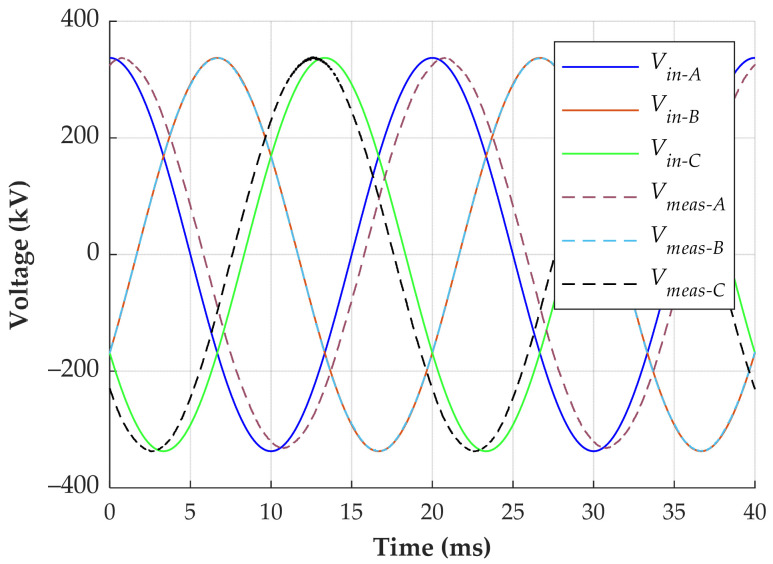
Comparison between the reference and measured voltages.

The first calibration method compares the voltage values measured by the CDSs with the AC voltage from a substation divider at 50 Hz. In this case, the primary voltage measured by a capacitive voltage transformer serves as the reference. During the installation, the RMS value of the primary phase voltage was 238.45 kV, assuming a balanced three-phase system. The RMS voltages measured by the CDSs for phases A, B, and C were 2.03 V, 1.68 V, and 1.89 V, respectively. Therefore, the calculated voltage ratios for devices MD1, MD2, and MD3 are 117,461:1, 141,932:1, and 126,162:1, respectively. The voltage ratios can be set directly in the power quality monitor. Figure 13 shows the reference primary voltage (*V_in_*) and the measured voltage (*V_meas_*) considering the calculated voltage ratio.

In phase B, the reference and measured voltages are in phase. It happens due to the symmetrical influence of phases A and C on the device installed in phase B. However, the measured voltage of phase A is delayed by 14.14° with respect to the reference voltage of the same phase due to the influence of phases B and C. Similarly, the measured voltage of phase C leads the reference voltage by 13.12° due to the influence of phases A and B.

This calibration method is generally suitable for measuring steady-state voltages. It is straightforward to implement directly in the power quality monitor. However, the coupling between the measurement devices and adjacent phases may introduce notable errors when measuring electromagnetic transients, as will be shown in Section 5.

### 4.2. Compensation of the Coupling Capacitances

Given the constraints of the simplified method, especially in measuring transient overvoltages, a post-processing calibration method is developed. This method considers the coupling capacitances between the sensors and the high-voltage connections of the three phases to reconstruct the primary voltages from the measured output voltages.

This calibration method also requires the information of the steady-state primary voltage during the installation of the measurement system. The same primary voltage measured by a CVT and the corresponding output signals of the CDSs are used. The primary voltages are considered symmetrical, with the same amplitude and a phase difference of 120°. Therefore, the primary phase voltages are expressed in phasor notation as:

(3) $$V_{1A}=V_{p}\;∠\;0{}^{\circ}\;V_{1B}=V_{p}\;∠\;120{}^{\circ}\;V_{1C}=V_{p}\;∠-120{}^{\circ}$$

where *V_p_* is the peak voltage measured by the CVT, in this case 337.22 kV.

**Figure 14:**
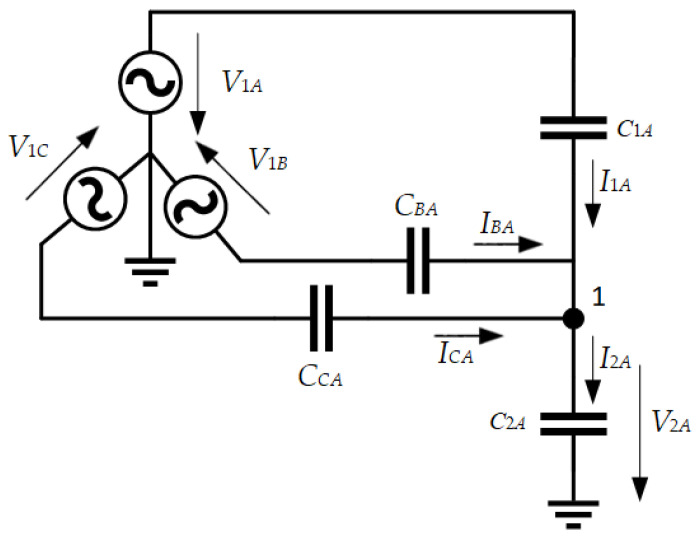
Equivalent circuit representing the measurement device installed in phase A.

Given the schematic diagram shown in Figure 12, an equivalent circuit for the measurement device installed in phase A is established to calculate the output voltage as a function of the primary voltage. The resulting equivalent circuit is presented in Figure 14.

Applying Kirchhoff’s current law for node 1, the current *I*_2*A*_ can be calculated as:

(4) $$I_{2A}=I_{1A}+I_{BA}+I_{CA}$$

where

(5) $$I_{2A}=j\omega C_{2A}\cdot V_{2A}\;I_{1A}=j\omega C_{1A}\cdot (V_{1A}-V_{2A})\;I_{BA}=j\omega C_{BA}\cdot (V_{1B}-V_{2A})\;I_{CA}=j\omega C_{CA}\cdot (V_{1C}-V_{2A})$$

Substituting the equations from (5) into Equation (4), the output voltage *V*_2*A*_ is calculated as:

(6) $$V_{2A}=\frac{1}{\left(C_{2A}+C_{1A}+C_{BA}+C_{CA}\right)}(C_{1A}\cdot V_{1A}+C_{BA}\cdot V_{1B}+C_{CA}\cdot V_{1C})$$

The capacitance *C*_2*A*_ has a value of approximately 50 nF, which is much higher than the stray capacitances *C*_1*A*_, *C_BA_*, and *C_CA_*, usually less than 1 pF. Therefore, Equation (6) can be simplified as:

(7) $$V_{2A}=\frac{1}{C_{2A}}(C_{1A}\cdot V_{1A}+C_{BA}\cdot V_{1B}+C_{CA}\cdot V_{1C})$$

The same concept and simplifications can be applied to the devices installed in phases B and C. Then, the output voltages are calculated as:

(8) $$\left[\begin{array}{c} V_{2A}\\ V_{2B}\\ V_{2C} \end{array}\right]=\left[\begin{array}{ccc} \frac{C_{1A}}{C_{2A}} & \frac{C_{BA}}{C_{2A}} & \frac{C_{CA}}{C_{2A}}\\ \frac{C_{AB}}{C_{2B}} & \frac{C_{1B}}{C_{2B}} & \frac{C_{CB}}{C_{2B}}\\ \frac{C_{AC}}{C_{2C}} & \frac{C_{BC}}{C_{2C}} & \frac{C_{1C}}{C_{2C}} \end{array}\right]\left[\begin{array}{c} V_{1A}\\ V_{1B}\\ V_{1C} \end{array}\right]$$

Equation (8) describes a linear system wherein the coefficient matrix depends on the stray capacitances and the user-defined capacitances *C*_2_. Since the stray capacitances have a unique value for a particular layout, there is only one physical solution for this system. However, all stray capacitances are unknown, leading to nine unknown parameters. The symmetry in the sensor installation reduces the number of unknown parameters, yet even in this scenario, the system may not be entirely determined. 

Assuming identical influences of phases A and C on the sensor installed in phase B, *C_AB_* is equal to *C_CB_*, and *V*_2*B*_ has the same phase as *V*_1*B*_ in steady-state voltage calibration. Additionally, the influence of phase B on the sensors in phases A and C is assumed to be the same, resulting in *C_BA_* being equal to *C_BC_*. The summarized assumptions are as follows:

(9) $$C_{BA}=C_{AB}=C_{CB}=C_{BC}$$

As previously stated, the linear system has mathematically infinite solutions, even with the assumptions outlined in (9). One potential approach, explored in other works [30,31,32], is to neglect the coupling capacitance between the outer phases, i.e., to consider *C_CA_* and *C_AC_* equal to 0. However, this choice may result in substantial errors when evaluating transient overvoltages. An alternative is to assign values obtained from FEM simulation to these parameters. Upon defining *C_CA_*, all parameters can be calculated by formulating the equations presented in (8), considering the assumptions given in (9).

The measurement system is calibrated using five distinct values of *C_CA_*, ranging from 0 to 0.2 pF, to assess the impact of coupling between the outer phases. The values have been chosen so that the calculated parameters are of the same order of magnitude as those obtained in the FEM simulation. The calculated parameters are shown in Table 2.

**Figure 15:**
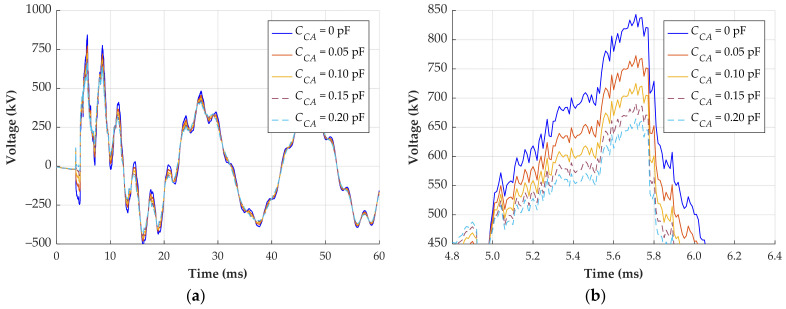
Transient overvoltage reconstruction with different calibration parameters. (**a**) Response of phase C; (**b**) zoomed waveforms on the region of maximum overvoltage.

A transient overvoltage is reconstructed using the five parameter sets from Table 2. Figure 15 shows the signals from phase C, where the maximum overvoltage occurred.

In the scenario where *C_CA_* is not considered (parameter set 1), the reconstructed signal exhibits a maximum overvoltage of 843 kV. Conversely, when *C_CA_* is 0.20 pF (parameter set 5), the reconstructed signal reaches only 665 kV. This difference of 178 kV represents 21.1%. Therefore, neglecting the coupling capacitance between the outer phases may lead to substantial errors in reconstructing the primary voltage. The question that arises is how to compute the correct coupling capacitances.

Based on the established principle of parallel plate capacitors, some configurations yield a capacitance inversely proportional to the distance between the electrodes. Hence, the initial investigation aimed to establish this correlation for the specific layout where the measurement system was installed. However, this relationship could not be established. 

Then, an investigation is conducted to determine if the stray capacitances exhibit an inverse proportionality to the square of the distance, represented as follows:

(10) $$C_{1A}\propto \frac{1}{{d_{AA}}^{2}}\;C_{BA}\propto \frac{1}{{d_{BA}}^{2}}\;C_{CA}\propto \frac{1}{{d_{CA}}^{2}}$$

where *d_AA_*, *d_BA_*, and *d_CA_* are the distances from the CDS installed in phase A to the high-voltage connections of phases A, B, and C, respectively.

Based on the information presented in (10) and considering that the permittivity and electrode area are identical for all phases, three normalized ratios can be calculated:

(11) $$\frac{C_{1A}}{C_{BA}}={\left(\frac{d_{BA}}{d_{AA}}\right)}^{2}\;∴\;\frac{C_{1A}}{C_{BA}}.{\left(\frac{d_{AA}}{d_{BA}}\right)}^{2}=1\;\frac{C_{BA}}{C_{CA}}={\left(\frac{d_{CA}}{d_{BA}}\right)}^{2}\;∴\;\frac{C_{BA}}{C_{CA}}.{\left(\frac{d_{BA}}{d_{CA}}\right)}^{2}=1\;\frac{C_{1A}}{C_{CA}}={\left(\frac{d_{CA}}{d_{AA}}\right)}^{2}\;∴\;\frac{C_{1A}}{C_{CA}}.{\left(\frac{d_{AA}}{d_{CA}}\right)}^{2}=1$$

**Figure 16:**
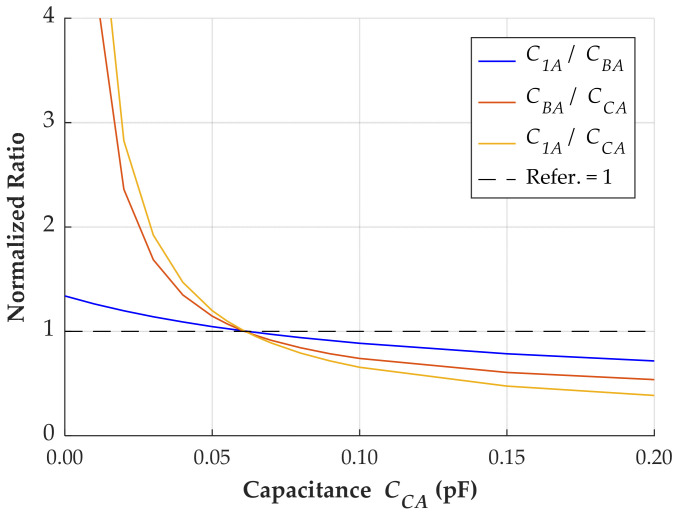
Normalized ratios according to different values of stray capacitances.

If the assumption that the capacitance is inversely proportional to the square of the distance is accurate, a set of stray capacitances satisfying the equations in (11) can be identified. Figure 16 shows the normalized ratios for different parameters, represented by the stray capacitance *C_CA_*.

When the capacitance *C_CA_* is 0.061 pF, the three normalized ratios become equal to 1. It indicates that a specific set of parameters satisfies the three equations given in (11). The parameters for the layout of this case study are presented in Table 3.

The correlation between the stray capacitances and the square of the distance has been demonstrated for the layout where the measurement system was installed. However, further investigation is necessary to confirm its validity for other configurations. If this correlation cannot be clearly established, alternative solutions include neglecting the coupling capacitance between the outer phases or estimating it using FEM simulations.

## 5. Measurement Results

The measurement system has been installed in the substation for one year, recording transients associated with transmission line switching and other disturbances. This section outlines the reconstruction of three transient signals using the calibration methods discussed in Section 4 and provides a statistical analysis of the recorded transients.

### 5.1. Comparison of Calibration Methods 

As detailed in Section 4.1, the calibration provided by the simplified method is appropriate for measuring steady-state voltages. However, it may lead to significant errors when reconstructing transient signals. Therefore, the recommended approach is to utilize the method outlined in Section 4.2, which compensates for coupling capacitances. Nevertheless, calculating the coupling capacitances can be challenging, and a simplification can be made by considering the coupling capacitance between the outer phases as neglectable.

To assess the dissimilarities in the reconstruction of real transient signals, three transients are evaluated using the following methods and parameters:

- Simplified method (SM);
- Compensation of coupling capacitances (CCC) with *C_CA_* = 0 pF;
- Compensation of coupling capacitances (CCC) with *C_CA_* = 0.061 pF.

**Figure 17:**
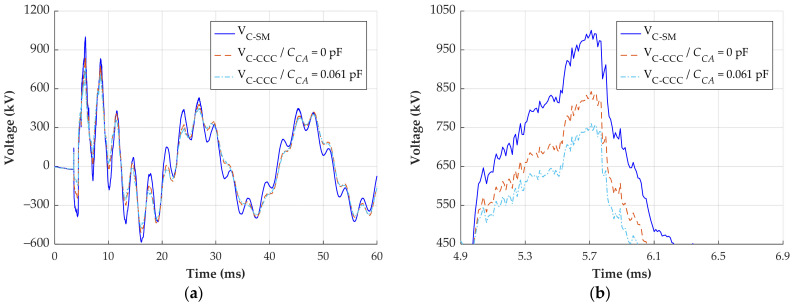
Transient overvoltage reconstruction—line energization. (**a**) Response of phase C; (**b**) zoomed waveforms on the region of maximum overvoltage.

The first transient (also shown in Section 4) occurs due to the energization of the transmission line. Figure 17 shows the reconstructed signals for phase C (most critical).

The signal reconstructed using the CCC method with a *C_CA_* of 0.061 pF is considered the reference. The maximum voltage of this signal is 761 kV. Ignoring the *C_CA_* in the CCC method results in a maximum voltage of 843 kV, representing a difference of 82 kV or 10.8%. The signal reconstructed with the simplified method has a maximum voltage of 1000 kV, indicating a difference of 31.4%.

**Figure 18:**
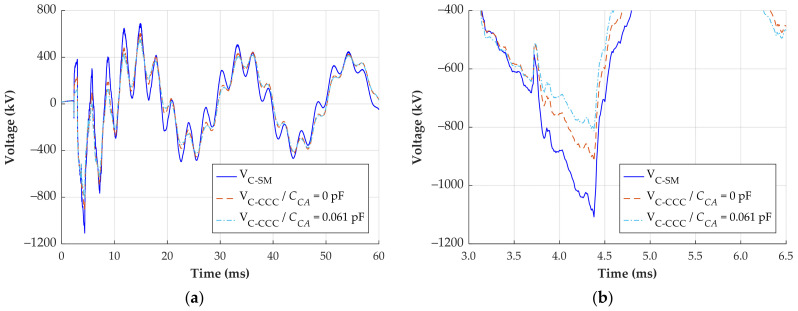
Transient overvoltage reconstruction—line energization. (**a**) Response of phase C; (**b**) zoomed waveforms on the region of maximum negative overvoltage.

The second signal is also a transient that occurs during the energization of the transmission line. The signal reconstruction of phase C, where the maximum negative overvoltage happened, is shown in Figure 18.

Considering again the signal reconstructed using the CCC method with a *C_CA_* of 0.061 pF as reference, the maximum negative voltage is −813 kV. Employing the CCC method with neglected *C_CA_* results in a maximum negative voltage of −916 kV, representing a difference of 103 kV or 12.7%. The signal reconstructed using the simplified method has a maximum negative voltage of −1107 kV, representing a difference of 294 kV or 36.2%. Therefore, the simplified method evidently overestimates the maximum overvoltage of the analyzed transients, whereas the difference ranging from 10% to 13% when ignoring *C_CA_* may or may not be considered acceptable, depending on the evaluation’s objective.

**Figure 19:**
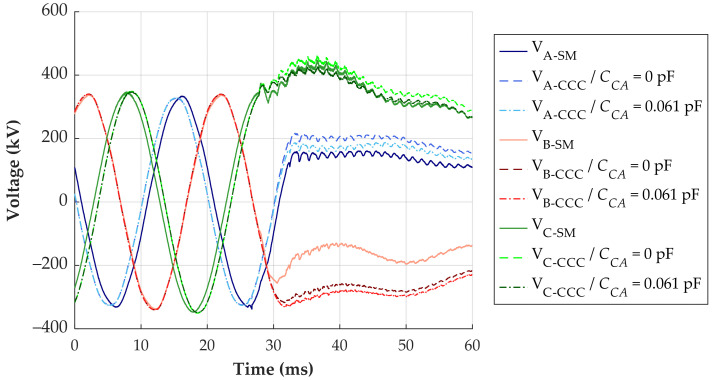
Transient overvoltage reconstruction—line de-energization.

The third evaluated transient was recorded during the de-energization of the transmission line. The reconstructed signals are shown in Figure 19. 

The most significant difference between the reconstructed signals is observed in phase B. The simplified method indicates a difference of up to 148 kV compared to the CCC method with a *C_CA_* of 0.061 pF. Moreover, the CCC method with neglected *C_CA_* and CCC method with a *C_CA_* of 0.061 pF have a maximum difference of approximately 30 kV. For this signal, no critical voltage level for equipment operation is identified. Consequently, the utilization of the simplified method would not compromise the evaluation.

### 5.2. Statistical Analysis of Transient Signals 

During the one-year measurement campaign, the measurement system recorded 142 transient events. Table 4 shows the classification of these transients.

Sixty-one percent of all recorded transients originate from external disturbances, such as circuit switching in the same substation or other perturbations in the power system. These signals are transients that return to the previous steady-state within a few milliseconds, meaning that there is no change in the state (closed/open) of the transmission line. The transmission line underwent 28 de-energizations and 27 energizations during the one-year period. This difference occurs because the line was energized when the measurement system was installed and de-energized when the system was removed.

**Figure 20:**
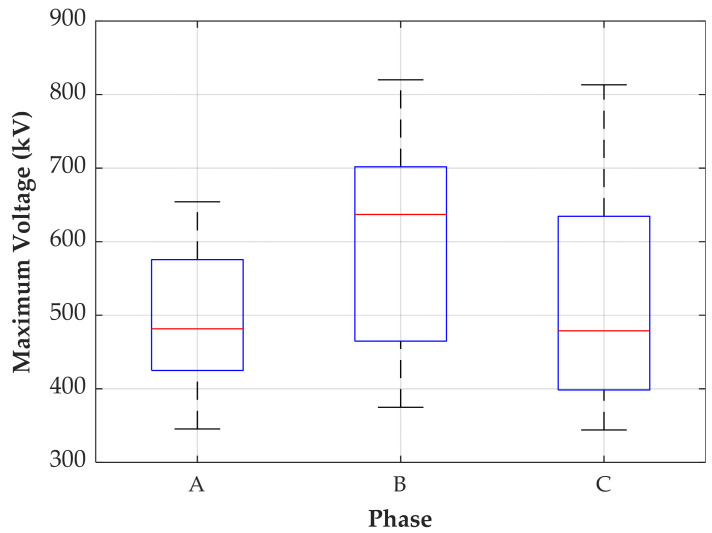
Maximum voltage measured during the energization of the transmission line.

The most critical transient overvoltages occurred during the energization of the transmission line. Therefore, a statistical analysis of the maximum voltage of the recorded transients is performed and the results are presented in Figure 20.

**Figure 21:**
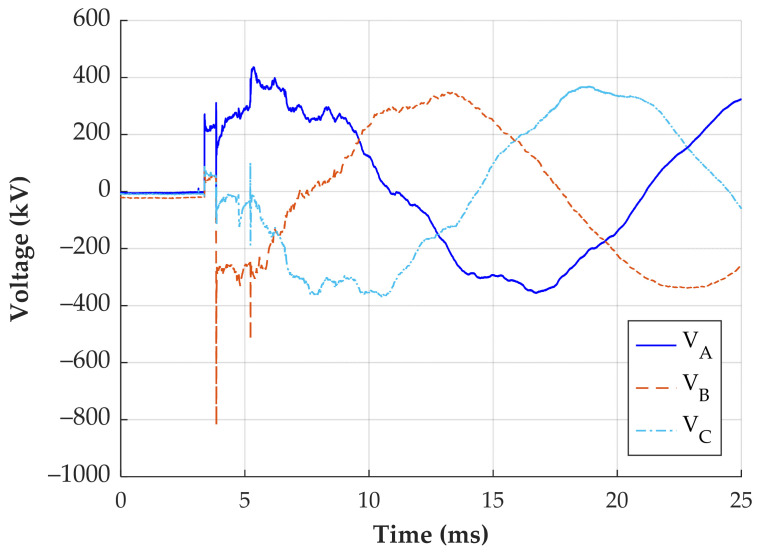
Transient signal with the maximum measured overvoltage.

The median of the maximum voltage registered for phases A, B, and C are 482 kV, 637 kV, and 479 kV, respectively. The maximum overvoltages are 654 kV, 820 kV, and 813 kV, respectively. Therefore, the maximum registered overvoltage, measured in phase B, represents about 2.4 times the rated peak voltage. Figure 21 shows the transient signal with the highest maximum (negative) overvoltage.

## 6. Conclusions

Accurately measuring and investigating electromagnetic transients is gaining importance with the increasing integration of renewable energy sources into the power grid. This paper introduces a measurement system based on capacitive electric field sensors capable of accurately measuring fast transients due to its broadband response. The measurement system was installed in a high-voltage substation for one year, recording 142 transient events. The installation process is straightforward and does not require de-energizing the bay.

Calibration is essential for measuring transient overvoltages using the proposed measurement system. This paper discusses two techniques: the simplified method and the CCC method. The simplified method can measure steady-state voltages, but it has been proven inaccurate for measuring transients. On the other hand, the CCC method, which accounts for the coupling capacitances between the measurement devices and the three phases, is the most appropriate for reconstructing transient overvoltages. All coupling capacitances were successfully calculated based on the layout and simplifications. However, neglecting the coupling capacitance between the external phases is acceptable if no relation between the layout (distances) and the calculated capacitances can be established. This study found that neglecting it resulted in a difference between 10% and 13%.

Finally, the study demonstrates that the proposed measurement system serves as an efficient and flexible solution for the long-term monitoring of transient overvoltages in a high-voltage substation, especially for fast transients requiring broadband measurement.

## Figures and Tables

**Table 1:** Ratio error considering the average of the measured voltage ratios.

Device MD1			Device MD2			Device MD3		
Input Voltage *V_in_* (kV)	Meas. Voltage *k_r-MD_*_1_ *V_out_* (kV)	Ratio Error *ε* (%)	Input Voltage *V_in_* (kV)	Meas. Voltage *k_r-MD_*_2_ *V_out_* (kV)	Ratio Error *ε* (%)	Input Voltage *V_in_* (kV)	Meas. Voltage *k_r_*_−*MD*3_ *V_out_* (kV)	Ratio Error *ε* (%)
13.92	13.90	−0.15	13.16	13.16	0.03	13.46	13.49	0.17
20.20	20.19	−0.03	20.69	20.75	0.29	20.72	20.69	−0.13
28.09	28.01	−0.28	28.61	28.69	0.26	27.82	27.82	0.02
35.80	35.91	0.29	34.58	34.48	−0.28	35.19	35.30	0.33
41.31	41.43	0.28	41.31	41.20	−0.26	41.31	41.38	0.16
47.12	47.08	−0.08	47.43	47.50	0.15	47.43	47.59	0.34
53.55	53.65	0.19	55.39	55.30	−0.16	54.47	54.37	−0.18
60.59	60.50	−0.15	62.12	62.02	−0.16	62.42	62.14	−0.46
67.63	67.62	0.00	68.54	68.60	0.08	68.85	68.63	−0.32
76.19	76.15	−0.06	76.50	76.54	0.05	76.19	76.26	0.08

**Table 2:** Set of stray capacitances calculated from different *C_CA_* values.

Param. Set No.	*C_CA_* (pF)	*C_AC_* (pF)	*C*_1*A*_ (pF)	*C*_1*B*_ (pF)	*C*_1*C*_ (pF)	*C_BA_* (pF)	*C_AB_* (pF)	*C_CB_* (pF)	*C_BC_* (pF)
1	0.000	0.018	0.476	0.473	0.452	0.121	0.121	0.121	0.121
2	0.050	0.068	0.526	0.523	0.502	0.171	0.171	0.171	0.171
3	0.100	0.118	0.576	0.573	0.552	0.221	0.221	0.221	0.221
4	0.150	0.168	0.626	0.623	0.602	0.271	0.271	0.271	0.271
5	0.200	0.218	0.676	0.673	0.652	0.321	0.321	0.321	0.321

**Table 3:** Set of stray capacitances calculated for the specific layout.

*C_CA_* (pF)	*C_AC_* (pF)	*C*_1*A*_ (pF)	*C*_1*B*_ (pF)	*C*_1*C*_ (pF)	*C_BA_* (pF)	*C_AB_* (pF)	*C_CB_* (pF)	*C_BC_* (pF)
0.061	0.079	0.537	0.534	0.513	0.182	0.182	0.182	0.182

**Table 4:** Number of transient events recorded by the measurement system.

Line Energization (No. of Events)	Line De-Energization (No. of Events)	External Disturbance (No. of Events)	Total
27	28	87	142

## Data Availability

Data are contained within the article.
